# Changes in the Treatment Responses to Artesunate-Mefloquine on the Northwestern Border of Thailand during 13 Years of Continuous Deployment

**DOI:** 10.1371/journal.pone.0004551

**Published:** 2009-02-23

**Authors:** Verena Ilona Carrara, Julien Zwang, Elizabeth A. Ashley, Ric N. Price, Kasia Stepniewska, Marion Barends, Alan Brockman, Tim Anderson, Rose McGready, Lucy Phaiphun, Stephane Proux, Michele van Vugt, Robert Hutagalung, Khin Maung Lwin, Aung Pyae Phyo, Piyanuch Preechapornkul, Mallika Imwong, Sasithon Pukrittayakamee, Pratap Singhasivanon, Nicholas J. White, François Nosten

**Affiliations:** 1 Shoklo Malaria Research Unit, Tak, Thailand; 2 Faculty of Tropical Medicine, Mahidol University, Bangkok, Thailand; 3 Centre for Clinical Vaccinology and Tropical Medicine Churchill Hospital, Headington, Oxford, United Kingdom; 4 Menzies School of Health Research, Charles Darwin University, Darwin, Australia; 5 Southwest Foundation for Biomedical Research, San Antonio, Texas, United States of America; University of California Los Angeles, United States of America

## Abstract

**Background:**

Artemisinin combination treatments (ACT) are recommended as first line treatment for falciparum malaria throughout the malaria affected world. We reviewed the efficacy of a 3-day regimen of mefloquine and artesunate regimen (MAS_3_), over a 13 year period of continuous deployment as first-line treatment in camps for displaced persons and in clinics for migrant population along the Thai-Myanmar border.

**Methods and Findings:**

3,264 patients were enrolled in prospective treatment trials between 1995 and 2007 and treated with MAS_3_. The proportion of patients with parasitaemia persisting on day-2 increased significantly from 4.5% before 2001 to 21.9% since 2002 (*p*<0.001). Delayed parasite clearance was associated with increased risk of developing gametocytaemia (AOR = 2.29; 95% CI, 2.00–2.69, *p* = 0.002). Gametocytaemia on admission and carriage also increased over the years (*p* = 0.001, test for trend, for both). MAS_3_ efficacy has declined slightly but significantly (Hazards ratio 1.13; 95% CI, 1.07–1.19, *p*<0.001), although efficacy in 2007 remained well within acceptable limits: 96.5% (95% CI, 91.0–98.7). The in vitro susceptibility of *P. falciparum* to artesunate increased significantly until 2002, but thereafter declined to levels close to those of 13 years ago (geometric mean in 2007: 4.2 nM/l; 95% CI, 3.2–5.5). The proportion of infections caused by parasites with increased *pfmdr1* copy number rose from 30% (12/40) in 1996 to 53% (24/45) in 2006 (*p* = 0.012, test for trend).

**Conclusion:**

Artesunate-mefloquine remains a highly efficacious antimalarial treatment in this area despite 13 years of widespread intense deployment, but there is evidence of a modest increase in resistance. Of particular concern is the slowing of parasitological response to artesunate and the associated increase in gametocyte carriage.

## Introduction

The Thai-Myanmar border harbors some of the world's most drug resistant malaria parasites. *Plasmodium falciparum* parasites in this region have developed significant resistance to commonly used antimalarials, except the artemisinin derivatives. The treatment of uncomplicated *P. falciparum* malaria in Thailand has been modified several times during the past 30 years to counter the rapid emergence and spread of drug resistance [1], [2]. A two-day combination of mefloquine and artesunate, MAS_2_, (artesunate 6 mg/kg repeated once 24 hours later), was introduced in Thailand in 1995, under strict supervision, exclusively in three provinces (including the northwestern border of Thailand) where failure rates with mefloquine (15 mg/kg) -sulfadoxine-pyrimethamine (MSP) had risen above 50% [3]. In 2004, MAS_2_ was extended to all provinces along the borders with Myanmar and Cambodia, following evidence from these areas of inadequate clinical cure rates with mefloquine alone, or MSP [4]–[6]. Use of both drugs has been strictly controlled and neither mefloquine nor artesunate are available in the private sector on the Thai side of the border.

In camps for displaced persons located along the Thai-Myanmar border, mefloquine and artesunate combination therapy has been evaluated since 1991 and deployed systematically since 1994 [7], [8]. The total dose of artesunate (12 mg/kg) was the same as that in the National protocol, but the duration of the treatment was 3 days (4 mg/kg/day) i.e. MAS_3_, to ensure that artesunate covered two parasite asexual life-cycles, thereby reducing the parasite biomass exposed to mefloquine alone [9]. MAS_3_, one of the ACT regimens currently recommended by the WHO, is also used in clinics serving the migrant population, located along the Thai-Myanmar border, and since January 2008 has replaced MAS_2_ in the Thailand National protocol.

This report presents a continuous description of the in vivo, in vitro and molecular correlates of MAS_3_ efficacy in the thirteen years between January 1995 and December 2007 in camps for displaced people and clinics for migrant workers located along the northwestern border of Thailand.

## Methods

### Study area

The epidemiology of malaria in the area has been described in detail elsewhere [10]. Briefly, malaria is seasonal, the transmission is low and unstable, *P. falciparum* and *P. vivax* are the two predominant species, *P. malariae* is occasionally found, and *P. ovale* is rare. All age groups are affected and nearly all the *P. falciparum* infections are symptomatic. Since 1995, the Shoklo Malaria Research Unit (SMRU) has conducted 7 prospective randomized open-label controlled chemotherapeutic trials of new antimalarial drugs in which MAS_3_ was one of the treatment arms [11]–[17] and two non-comparative trials of MAS_3_ drug efficacy [18].

### Assessment of treatment regimens

Three methodologies were use to evaluate MAS_3_ efficacy: in vivo, in vitro and molecular assessment of *pfmdr1* copy number. The in vivo efficacy of MAS_3_ has been monitored annually [18], [19]. The prospective analysis of in vivo efficacy was restricted to studies after 1994, when distinction of recrudescent from novel infections was made possible by the introduction of PCR parasite genotyping [20], thereby ensuring accurate assessment of recrudescence rates. Post-treatment follow-up was extended to 42–63 days to minimize underestimation of true failure rates [21], [22].

In vitro antimalarial drug susceptibility studies were carried out on fresh isolates of *P. falciparum* parasites from 1995 onwards [23]. Studies in this location have demonstrated that amplification of *pfmdr1* gene copy number is the major molecular determinant of mefloquine susceptibility and provides an important tool for monitoring drug resistance [24], [25].

### In vivo studies of drug efficacy

#### Study procedures

Specific study procedures have been described previously for each randomized controlled trial [11]–[17]; most enrolment procedures were common to all studies. In brief patients, who presented to the SMRU clinics (all located along 100 km of the Thai-Myanmar border) with a microscopically confirmed uncomplicated *P. falciparum* malaria or a mixed infection, were enrolled after giving written (all randomized trials and all non-comparative trials since 1996) or witnessed verbal consent (non-comparative trials conducted in 1995). Severely ill patients and hyperparasitaemic patients (4% or more infected red blood cells on a thin malaria smear) were treated with different regimens and were not included in this analysis. Patients who had received treatment with mefloquine in the previous 63 days or who had failed their last antimalarial drug treatment, pregnant women, children <5 kg in body weight, and patients with signs of severity or concomitant disease were excluded from the studies. All patients were examined daily until fever subsided (fever being defined as an axillary, rectal or tympanic temperature≥37.5°C) and until parasite clearance (defined as the absence of *P. falciparum* trophozoites on the malaria smear), and then followed weekly for 6 to 9 weeks. In the event of parasite reappearance during follow up patients were re-treated and followed-up again. The clinical trials conducted at SMRU were all reviewed by the Ethics Committee of the Faculty of Tropical Medicine Mahidol University and the Oxford Tropical Research Ethics Committee.

#### Drug regimens

Patients received oral artesunate (Guilin Pharmaceutical Factory, Guilin, Guangxi, People's Republic of China) or Arsumax® (Sanofi-Aventis, Gentilly-France) 4 mg/kg/day for 3 days once daily, and mefloquine 25 mg base/kg, Lariam® (Roche, Basel-Switzerland) or Mequin® (Atlantic Laboratories, Bangkok-Thailand). Children who were unable to swallow complete tablets received the same weight-adjusted dosage of crushed tablets mixed with water. Each drug administration was observed in all patients.

Patients received mefloquine either as a single dose of 25 mg/kg (during trials conducted before 1996), as a split dose of 15 mg/kg and 10 mg/kg given 24 hrs apart on the second and third day of treatment (the current protocol), or as a split dose of 8 mg/kg given once a day for 3 days in two trials [15], [16].

In the 2006 dose-assessment study [17], half of the patients received a newly developed fixed co-formulation of mefloquine HCl 220 mg (or mef 200)+artesunate 100 mg (adult tablet) and mefloquine HCl 55 mg (or mef 50)+artesunate 25 mg (paediatric tablet), (Far-Manguinhos, Brazil), given once a day for 3 days according to body weight. This fixed dose combination has been shown to have comparable oral bioavailability and efficacy with the loose combination [17].

#### Outcome measures

Recurrent infection was defined as the presence of asexual forms of *P. falciparum* parasite on a blood smear between day-5 and 45 of follow-up (42 days+3 days to take into account a delay in the last follow-up). Recrudescent infections were differentiated from new infections using 3 loci genotyping as described previously [20]. Patients with recurrent infection were usually treated with 7 days of artesunate (2 mg/kg/day), which was combined with 7 days of doxycycline 4 mg/kg/day (T.O. Chemicals Ltd, Bangkok-Thailand) in patients >8 years old.

In view of the importance of the parasite reduction ratio [26], delayed parasite clearance was considered if patients were still parasitaemic 48 hours (day-2) after the baseline malaria positive smear.

### In vitro studies of P. falciparum drug susceptibility

Parasites isolates were obtained from patients with acute *P. falciparum* malaria infection attending the research clinics. Only primary infections were assessed. Isolates were collected if the parasite density was at least 5 parasites/1,000 red blood cells (RBC), from patients attending the clinic, whether enrolled in one of the studies or not. Venous blood (5 ml) was collected into a sterile Vacutainer® tube containing 0.05 ml Potassium-EDTA. Samples were kept at room temperature and transported within the next 4–6 hours to the main laboratory, where they were set up in continuous culture immediately. The minimum parasitaemic threshold required for parasite growth in culture, as well as the necessity to take 5 ml of blood limited the evaluation of *P. falciparum* drug susceptibility to patients with higher parasitaemia who agreed to have a venopuncture (usually adults).

#### In vitro drug assay

Drug susceptibility testing by hypoxanthine uptake has been described previously by Brockman [23]. Briefly sodium artesunate (AS), and mefloquine (MFQ) were dissolved in 70% ethanol at a concentration of 1 mg/ml, and serial dilutions (683 ng/ml to 0.67 ng/ml for mefloquine and 33.46 ng/ml to 0.03 ng/ml for artesunate) were made in complete RPMI medium. The solvent in the final concentrations had no significant effect on parasite growth when compared to culture media. All concentrations, including drug-free controls, were distributed in duplicate in 96-well tissue culture plates. The drug-plates were made in bulk and stored at −80°C until use (for up to 3 months). The reproducibility of IC_50_ measurements was assessed regularly using cloned K1 isolates of *P. falciparum*.

### Molecular analysis of pfmdr1

DNA for molecular analysis of *pfmdr1* copy number was available from whole blood (in EDTA or heparinised tubes, stored at −70°C), or from 50 µl of capillary blood transferred to Whatman™ filter paper. In order to investigate changes in resistance markers over time parasite genotypes were assessed each year since 1996, with the exception of 1998, 2000 and 2007, using venous blood taken before treatment from a random selection of patients with primary *P. falciparum* infections. Randomisation was stratified by age and sex to represent the population seeking care for malaria in the outpatient's clinics.

*Pfmdr1* copy number was assessed by quantitative PCR (ABI sequence detector 7700; Applied Biosystems™) using the methods described previously [25]. All reactions were performed in triplicate and were rejected if they did not conform to exponential kinetics. Single nucleotide polymorphisms in *pfmdr1* were detected by nested PCR-RFLP methods as described [25]. All PCRs and digests included as positive and negative controls DNA of laboratory strains 3D7, HB3, 7G8 and Dd2, respectively.

### Statistical Analysis

Data were analyzed using SPSS for Windows™ (Version 14, SPSS Inc.) and STATA (Version 10, Stata Corp.). Differences in settings, sequence randomization, mefloquine treatment timing, and study procedures were included in the assessment of heterogeneity between studies using the Cochran Q test and the I2 test [27]. The cumulative risk of failure at day-42 was calculated by survival using the Kaplan Meier method. Indeterminate genotyping results and new infections were censored at the date of the reappearance of the *P. falciparum* parasites. PCR results were deemed indeterminant if only one of the three gene locus was amplified, or if analysis of genetic frequency of alleles in the population revealed a recrudescent pair as statistically insignificant [20]. The risks of treatment failure (PCR-adjusted) over time were compared by the Mantel-Haenszel log rank test. In the multivariate analysis, temporal trends in efficacy were assessed by using a Cox regression model after controlling for known risk factors such as age, parasitaemia at baseline, and mixed parasite infection. Non-adjusted and adjusted Hazard ratios (HR and AHR) and Odds ratios (OR and AOR) were also presented. A stepwise logistic regression was used to analyze the changes in delayed parasite clearance time, with the model including variables significantly associated to parasite clearance time in the univariate analysis (level of significance if *p*<0.10), as well as other potential risks factors such as age, sex, admission parasitaemia, mixed parasite infection, and type of drug regimen.

Gametocytaemia was considered as a binary variable (present/absent) and analyzed on admission, at day-1, 2, 3, 7, and weekly thereafter until day-42. Gametocyte carriage rates were presented as person-gametocyte-weeks (PGW, number of weeks patients had gametocytes/number of weeks patients were followed-up), and expressed per 1,000 weeks of follow-up. Prevalence rate on admission, overall carriage rate and among patients without gametocyte on admission were calculated yearly.

In vitro dose-response curves were analyzed by fitting the data to an inhibitory E-max pharmacokinetic model using WINNONLIN (Version 4.1, Pharsight Corporation) and the IC_50_ values, and coefficients of variation calculated. Temporal trends for mefloquine and artesunate IC_50_ were analyzed on the ln-transformed data.

Regression analysis on the ln-transformed values was performed to investigate association between the IC_50_ and the *pfmdr1* copy number using the isolates for which both the IC_50_ and the copy number were available.

Changes in *pfmdr1* copy number, gametocyte carriage rates, and the proportions of patients remaining febrile and/or parasitaemic on day 2–4 over the years were analyzed by χ^2^ test for trend.

## Results

The efficacy of the MAS_3_ combination was evaluated in 3,264 patients with uncomplicated *P. falciparum* malaria infections, enrolled in 9 trials, between 1995 and 2007 (**Table 1**). Heterogeneity between studies was low and was not significant (I^2^ test = 34%, *p* = 0.109, Cochran Q test).

**Table 1:** Patients enrolled in antimalarial drug efficacy trials who received MAS_3_, between 1995 and 2007, on the northwestern border of Thailand.

		1995	1996	1997	1998	1999	2000	2001	2002	2003	2004	2005	2006	2007
**Randomized Controlled Trials**														
Van Vugt et al. 1998	N = 305	31	274											
Van Vugt et al. 2000	N = 43			29	14									
Van Vugt et al. 2002	N = 534				156	280	98							
Hutagalung et al. 2005	N = 245							160	85					
Ashley et al. 2004&05	N = 343								127	210	6			
Ashley et al. 2006	N = 500										187	313		
Unpublished	N = 240											17	134	89
**Sub-total**	**N = 2210**	**31**	**274**	**29**	**170**	**280**	**98**	**160**	**212**	**210**	**193**	**330**	**134**	**89**
**Open trials for routine monitoring of MAS_3_**														
Price et al. 1997	N = 453	451	2											
Unpublished	N = 601							246		42	145	70	49	49
**Sub-total**	**N = 1054**	**451**	**2**	**0**	**0**	**0**	**0**	**246**	**0**	**42**	**145**	**70**	**49**	**49**
**Total**	**N = 3264**	**482**	**276**	**29**	**170**	**280**	**98**	**406**	**212**	**252**	**338**	**400**	**183**	**138**

The admission characteristics of patients are presented in **Table 2**. Overall 36% (1,159/3,264) of the patients enrolled were children under 15 years. In adults, there was a preponderance of males (73%, 1,533/2,105). Overall 19% (*n* = 625) of patients did not complete the 42-day follow-up period. The main reasons for loss of follow-up were change of address and employment obligations (592/625). The drug combination was generally well tolerated and few patients reported having serious adverse events [11]–[18]. Only one patient (0.03%; 95% CI, 0.0–0.2) deteriorated after starting MAS_3_ and required parenteral treatment; three deaths were reported (0.09%; 95% CI, 0.02–0.31). None were related to malaria or to an adverse drug event. There were no significant differences in age, sex and presence of mixed infections on admission between patients lost to follow up and those who completed it.

**Table 2:** Demographic characteristics on admission of patients enrolled in antimalarial drug efficacy trials who received MAS_3_, between 1995 and 2007.

	1995	1996	1997	1998	1999	2000	2001	2002	2003	2004	2005	2006	2007	Total
No enrolled	482	276	29	170	280	98	406	212	252	338	400	183	138	3264
No of males (%)	291 (60%)	190 (69%)	26 (90%)	126 (74%)	183 (65%)	67 (68%)	258 (64%)	142 (67%)	153 (61%)	215 (64%)	283 (71%)	145 (79%)	121 (88%)	2200 (67%)
Age (years)														
<15 years	317 (65.8%)	92 (33.3%)	6 (20.7%)	50 (29.4%)	88 (31.4%)	38 (38.8%)	158 (38.9%)	80 (37.7%)	95 (37.7%)	107 (31.7%)	115 (28.8%)	9 (4.9%)	4 (2.9%)	1159 (35.5%)
≥15 years	165 (34.2%)	184 (66.7%)	23 (79.3%)	120 (70.6%)	192 (68.6%)	60 (61.2%)	248 (61.1%)	132 (62.3%)	157 (62.3%)	231 (68.3%)	285 (71.2%)	174 (95.1%)	134 (97.1%)	2105 (64.5%)
No with mixed infections (%)	86 (17.8%)	13 (4.7%)	1 (3.4%)	6 (3.5%)	20 (7.1%)	4 (4.1%)	36 (8.9%)	20 (9.4%)	23 (9.1%)	19 (5.6%)	30 (7.5%)	13 (7.1%)	2 (1.4%)	273 (8.4%)
Geometric mean parasitaemia (per µl)	6745	4932	7798	5984	3776	3228	7161	10544	10765	8375	7907	9226	7674	6982
Min	16	13	631	20	6	20	13	16	79	50	32	158	158	6
Max	199526	251189	125893	251189	501187	158489	199526	199526	199526	316228	316228	199526	158489	501187

### Clinical and parasitological responses

Changes in fever clearance times were evaluated in the 2,029 (62%) of patients who presented with fever (**Table 3**). Overall, 91.4% (95% CI, 90.1–92.6) of patients were afebrile by day-2, and 98.7% (95% CI, 98.0–99.1) by day-3 without significant time trend. Mean haematocrit on admission was 37.1% (95% CI, 36.9–37.3) and remained unchanged over the 13 year period. Mean fractional reductions in haematocrit from baseline at day-7 did not differ significantly by year (overall mean reduction of 7.4%; 95% CI, 6.9–7.9).

**Table 3:** Fever and parasite clearance time (in days).

	1995	1996	1997	1998	1999	2000	2001	2002	2003	2004	2005	2006	2007	Total
**Fever clearance**														
Febrile (N, %)														
Day 0 (admission)	406 (100)	169 (100)	20 (100)	104 (100)	170 (100)	44 (100)	301 (100)	118 (100)	87 (100)	179 (100)	256 (100)	131 (100)	44 (100)	2029 (100)
Day 1	199 (49.0)	68 (40.2)	7 (35.0)	35 (33.7)	65 (38.2)	14 (31.8)	141 (46.8)	29 (24.6)	18 (20.7)	55 (30.7)	90 (35.2)	64 (48.9)	18 (40.9)	803 (39.6)
Day 2	43 (10.6)	22 (13.0)	5 (25.0)	4 (3.8)	9 (5.3)	2 (4.5)	25 (8.3)	4 (3.4)	3 (3.4)	19 (10.6)	22 (8.6)	13 (9.9)	3 (6.8)	174 (8.6)
Day 3	5 (1.2)	6 (3.6)	2 (10.0)	1 (1.0)	1 (0.6)	0	1 (0.3)	0	1 (1.1)	5 (2.8)	4 (1.6)	0	1 (2.3)	27 (1.3)
Day 4	2 (0.5)	0	0	0	0	0	0	0	0	2 (1.1)	1 (0.4)	0	0	5 (0.2)
Day 5	1 (0.2)	0	0	0	0	0	0	0	0	0	1 (0.4)	0	0	2 (0.1)
**Parasite clearance**														
Smear positive (N, %)														
Day 0 (admission)	468 (100)	273 (100)	29 (100)	168 (100)	279 (100)	98 (100)	402 (100)	211 (100)	252 (100)	337 (100)	393 (100)	182 (100)	126 (100)	3218 (100)
Day 1	189 (40.4)	106 (38.8)	20 (69.0)	96 (57.1)	150 (53.8)	53 (54.1)	274 (68.2)	159 (75.4)	201 (79.8)	276 (81.9)	320 (81.4)	122 (67.0)	91 (72.2)	2057 (63.9)
Day 2	17 (3.6)	9 (3.3)	2 (6.9)	10 (6.0)	16 (5.7)	5 (5.1)	47 (11.7)	46 (21.8)	42 (16.7)	108 (32.0)	118 (30.0)	31 (17.0)	25 (20.0)	476 (14.8)
Day 3	0	0	0	1 (0.6)	1 (0.4)	0	8 (2.0)	8 (3.8)	13 (5.2)	19 (5.6)	26 (6.6)	3 (1.6)	1 (0.8)	80 (2.5)
Day 4	0	0	0	0	0	0	2 (0.5)	1 (0.5)	1 (0.4)	7 (2.1)	1 (0.3)	1 (0.6)	0	13 (0.4)
Day 5	0	0	0	0	0	0	0	0	0	1 (0.3)	1 (0.3)	0	0	2 (0.1)

**Figure 1:**
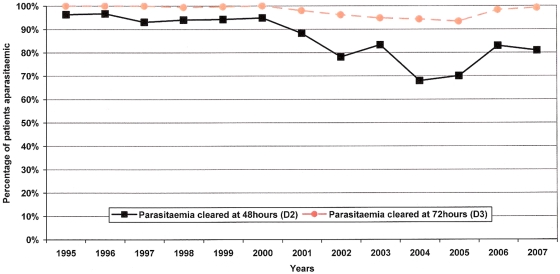
Percentage of patients who had cleared parasitaemia at Day-2 and Day-3 1995–2007.

Parasite clearance times were available for 3,218 patients (98.6%), (**Table 3**). There was a significant increase in the proportion of patients failing to clear their parasitaemia by day-2 over the study period (*p*<0.001, test for trend). Before 2001, 95.5% (1,256/1,315) were aparasitaemic within 48 hours compared to 78.1% (1,487/1,903) between 2001 and 2007 (OR = 5.96; 95% CI, 4.49–7.90, *p*<0.001). By day-3, 99.8% (1,313/1,315) patients had cleared their parasitaemia before 2001, but this fell to 95.9% (1,825/1,903) thereafter, (*p*<0.001), (**Figure 1**). Whereas until the end of 2000, no patients (0/1,315) were parasitaemic on day-4, between 2001 and 2007 there were 13 patients (0.7%) with parasites seen on day-4 (*p* = 0.007).

After accounting for age, sex, presence of a mixed (*P. falciparum*+*P. vivax*) infection, and haematocrit on admission, the increase in parasite clearance time over the 13 year study period remained significant (AOR = 1.11; 95% CI, 1.06–1.16, *p*<0.001) with a 0.05% annual increase of patients with delayed parasitaemia. In the same multivariate analysis, parasitaemia on admission (AOR = 2.47; 95% CI, 2.13–2.87, *p*<0.001), gametocytaemia on admission (AOR = 1.59; 95% CI, 1.07–2.37, *p* = 0.023), and site of recruitment (AOR = 0.32; 95% CI, 0.23–0.45, *p* = 0.001) were significantly associated with failure to clear parasitaemia within 48 hours.

### Gametocyte carriage

Overall 6.3% (196/3,109) of patients had patent gametocytaemia on admission and this proportion increased from 1.2% (6/482) in 1995 to 7.2% (10/139) in 2007 (*p* = 0.001, test for trend), resulting in a significant increase in gametocyte carriage rate over the study period (*p* = 0.001, test for trend), (**Table 4**). However, the gametocyte carriage rate among patients without gametocyte on admission remained unchanged throughout the study period (*p* = 0.30, test for trend).

**Table 4:** Gametocyte prevalence rate on admission and gametocyte carriage rate (overall and among patients without gametocytaemia on admission).

	1995	1996	1997	1998	1999	2000	2001	2002	2003	2004	2005	2006	2007
**Gametocytaemia on admission**													
Total number of patients	482	276	29	168	279	98	400	212	252	337	400	176	139
Prevalence (%)	1%	6%	7%	11%	8%	7%	3%	6%	5%	9%	10%	11%	7%
**Overall gametocyte carriage rate**													
Person-weeks of follow-up	1658	1052	143	641	988	382	2036	1148	1326	1817	2086	919	722
Gametocyte carriage (in weeks)	9	10	0.6	19	15	10	14	12	13	33	42	21	11
Gametocyte carriage rate (per 1000 PGW)	5	10	4	29	15	27	7	10	10	18	20	23	15
**Patients without gametocyte on admission**													
Person-weeks of follow-up	1569	939	127	554	888	336	1912	1048	1223	1419	1846	803	650
Gametocyte carriage (in weeks)	6	1	0	2	2	1	6	2	3	6	6	6	2
Gametocyte carriage rate (per 1000 PGW)	4	1	0	4	3	3	3	2	2	5	3	7	3

**Figure 2:**
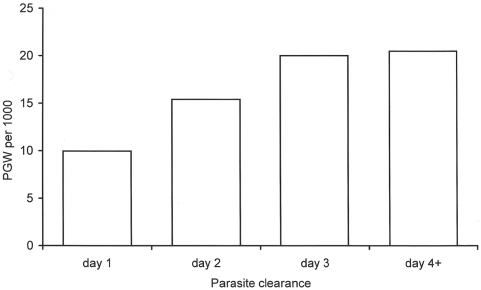
Relationship between parasite clearance time and gametocyte carriage. Gametocyte carriage rate is expressed per 1,000 person-gametocyte-week (PGW).

Patients who cleared their parasitaemia on day-1 had a lower gametocyte carriage (10 per 1,000 person-gametocyte-week (or PGW); 95% CI, 7–13) than those whose parasitaemia was cleared on day-2 (15 per 1,000 PGW; 95% CI, 13–18, *p* = 0.009), or on day-3 or later (20 per 1,000 PGW; 95% CI, 14–26, *p* = 0.051), (**Figure 2**).

The majority of patients without gametocytaemia on admission, who later developed gametocytaemia, did so on day-1 (43%). The proportion of patients with gametocyte appearance during follow-up did not increase significantly over the years (*p* = 0.082, test for trend), but was related to the parasite clearance time; it increased from 2.9% (74/2,580) among patients who cleared their parasitaemia on day-1 or on day-2 to 5.9% (26/437) among those who cleared their parasitaemia on day-3 or later, *p* = 0.001.

In the multivariate analysis, after controlling for gametocytaemia on admission (AOR = 1.65; 95% CI, 1.09–2.49, *p* = 0.018), parasitaemia on admission (AOR = 2.32; 95% CI, 2.00–2.69, *p* = 0.001), year of recruitment (AOR = 1.16; 95% CI, 1.11–1.22, *p* = 0.001), and site of recruitment (AOR = 0.37; 95% CI, 0.26–0.51, *p* = 0.001), patients who had a delayed parasite clearance (3 days or longer) remained at a higher risk of developing gametocytaemia (AOR = 2.29; 95% CI, 2.00–2.69, *p* = 0.002).

### Parasitological efficacy

Of the 333 recurrent infections with *P. falciparum* during the 42-day follow-up period, 177 (53%) were new infections, 108 (32%) were recrudescent infections, and in 48 (15%) cases the PCR result was inconclusive (*n* = 15) or samples were not available (*n* = 33). The median time to recrudescence occurred at 21 days of follow-up (range 7–42 days); although time to recrudescence has increased by one week since 2006, it has not changed significantly over the 13 year period, and neither has the time to re-infection (**Table 5**).

**Table 5:** PCR-confirmed novel and recrudescent infections and time to recrudescence.

	1995	1996	1997	1998	1999	2000	2001	2002	2003	2004	2005	2006	2007	Total
Total patients treated with MAS_3_	482	276	29	170	280	98	406	212	252	338	400	183	138	3264
Inconclusive (N)	0	0	0	0	1	1	4	0	0	2	4	3	0	15
Missing (N)	11	3	0	0	0	2	3	0	1	9	2	0	2	33
Novel infections (N)	11	5	0	1	7	13	29	12	13	23	30	25	8	177
Days to novel infection median, [range]	35 [21–64]	42 [35–63]	-	37	36 [28–42]	36.5 [20–49]	34.5 [14–63]	42 [21–63]	43.5 [14–63]	34 [13–61]	43 [14–70]	33 [21–63]	31.5 [14–50]	34.0 [13–45]
Recrudescent infections (N)	8	5	0	0	1	7	14	8	9	20	25	7	4	108
Days to recrudescence median, [range]	21 [14–41]	21 [16–36]	-	-	21	23 [15–33]	21 [14–42]	23.5 [21–42]	21 [7–28]	21 [15–42]	23 [14–42]	28 [21–42]	28 [23–34]	21 [7–42]

Parasitological efficacy (non PCR-adjusted) increased significantly over time as the risk of *P. falciparum* parasites recurrence within 42 days of follow-up, after controlling for age, sex, mixed parasite infections, gametocyte and parasitaemia on admission, decreased (AHR = 0.88; 95% CI, 0.86–0.91, *p* = 0.001).

**Figure 3:**
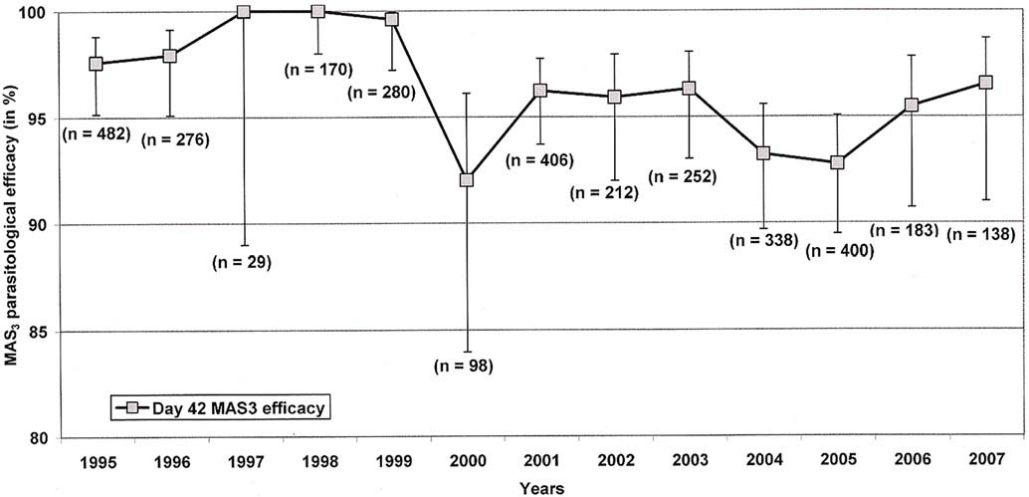
PCR-adjusted parasitological efficacy (95% CI) at Day-42 following MAS_3_ treatment. Parasitological efficacy (PCR-adjusted) of the mefloquine-artesunate 3-day combination therapy was evaluated at Day-42 of follow-up between 1995 and 2007. Efficacy is given as a percentage (95% CI).

A small but statistically significant decline in PCR-adjusted parasitological efficacy (assessed at day-42) was observed over the study period (HR = 1.13; 95% CI, 1.07–1.19, *p*<0.001). However the efficacy of MAS_3_ in 2007 remained above 95%: 96.5% (95% CI, 91.0–98.7), (**Figure 3**). The temporal trend of a slight decline in MAS_3_ efficacy remained after controlling for age, sex, baseline parasitaemia and gametocytaemia, mixed infections and the patients' enrolment location (AHR = 1.11; 95% CI, 1.02–1.21, *p* = 0.020).

### In vitro drug sensitivity

**Figure 4:**
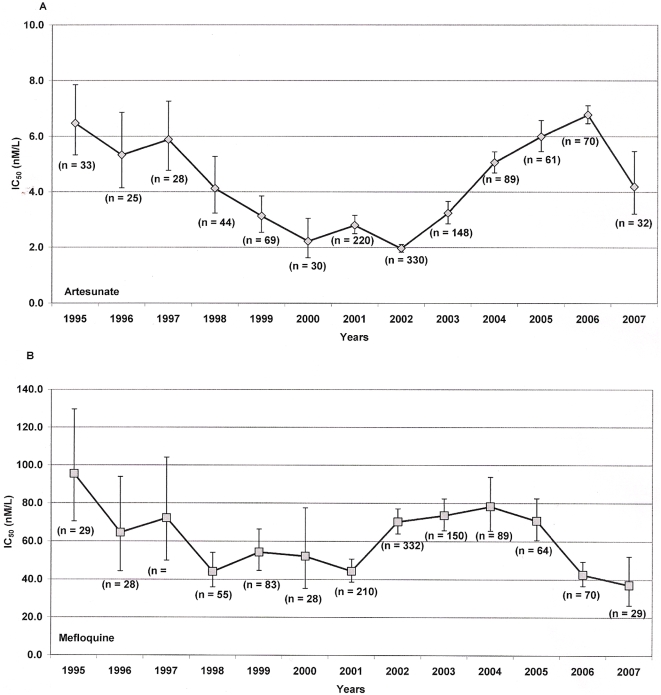
Drug sensitivity of *P. falciparum* isolates for artesunate (Figure 4a) and mefloquine (Figure 4b). Isolates from primary infections were collected at SMRU clinics between 1995 and 2007 and assayed for sensitivity to artesunate and mefloquine, IC_50_ geometric means are given as nM/l with [95% CI].

In total, 1,581 *P. falciparum* isolates were assayed for in vitro drug susceptibility during the study period; artesunate IC_50_ could be determined satisfactorily in 1,179 (75%) isolates and mefloquine IC_50_ in 1,195 (76%). Between 1995 and 2001 there was a significant decrease in the IC_50_s of both artesunate (13% per year, *p*<0.001) and mefloquine (9.1% per year, *p*<0.001). From 2002 this trend reversed for artesunate with IC_50_s increasing 41% per year (*p*<0.001) until 2006, although decreasing in 2007 (**Figure 4a**). Mefloquine IC_50_s also rose between 2001 and 2004, but fell again in 2006–7 (**Figure 4b**). Between 1995 and 2007, the geometric mean mefloquine IC_50_ fell from 95.5 nM/l (95% CI, 70.4–129.7) to 37.1 nM/l (95% CI, 26.5–52.3, *p*<0.001), and the IC_50_ of artesunate from 6.47 nM/l (95% CI, 5.34–7.86) to 4.19 nM/l (95% CI, 3.20–5.46, *p* = 0.009).

### *Pfmdr1* copy number

**Figure 5:**
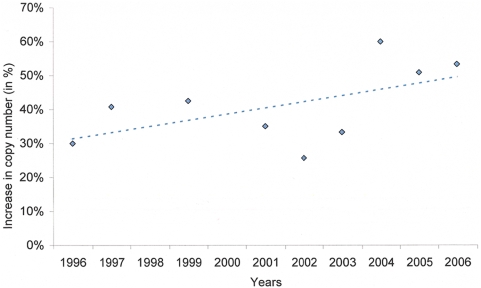
Proportion of primary infections with increased *Pfmdr1* (≥2) copy number. Isolates from primary infections were used for genetic analysis between 1996 and 2006. Increase in isolates with 2 or more copy number is given in percentage of the total.

A total of 531 isolates were selected randomly for genetic analysis with *pfmdr1* copy number successfully determined in 84% (444/531) of these isolates. The proportion of infections with two or more *pfmdr1* copy number rose from 30% (12/40) in 1996 to 53% (24/45) in 2006 (*p* = 0.012, test for trend), (**Table 6** **and** **Figure 5**).

**Table 6:** *Pfmdr1* copy number in parasite isolates collected in patients with primary uncomplicated *P. falciparum* infection.

	1996	1997	1999	2001	2002	2003	2004	2005	2006
Total isolates collected	50	53	60	68	76	46	61	67	50
Isolates with determination of *pfmdr1* copy number	40	49	47	57	70	36	45	55	45
*Pfmdr1* copy number:									
- 1	28 (70%)	29 (59%)	27 (58%)	37 (65%)	52 (74%)	24 (66%)	18 (40%)	27 (49%)	21 (47%)
- 2	8 (20%)	7 (14%)	11 (23%)	10 (18%)	12 (17%)	6 (17%)	13 (29%)	16 (29%)	11 (24%)
- ≥3	4 (10%)	13 (27%)	9 (19%)	10 (17%)	6 (9%)	6 (17%)	14 (31%)	12 (22%)	13 (29%)

Among 408 patients treated with MAS_3_ regimen for whom in vivo and molecular data were available, 183 (45%) were infected with parasites with increased *pfmdr1* copy number. Infections with increased *pfmdr1* copy number were at greater risk of recrudescence by day-42 compared to single copy number infections (AHR = 10.0; 95% CI, 3.0–33.3, *p*<0.001). There was no difference in the proportion of patients parasitaemic on day-2 in those with single (25%, 46/188) or increased *pfmdr1* copy number (22%, 34/155, *p* = 0.61), even after controlling for baseline parasitaemia.

In the 394 isolates with in vitro and molecular data, amplification of *pfmdr1* was associated with a 2.9 fold increase in mefloquine IC_50_ (geometric mean = 42.2 nM/l; 95% CI, 37.9–46.8 to 120.8 nM/l; 95% CI, 107.0–136.2, *p*<0.001) and a 1.7 fold increase in artesunate IC_50_ (1.99 nM/l; 95% CI, 1.81–2.19 to 3.33 nM/l; 95% CI, 2.94–3.80, *p*<0.001). The rise in IC_50_ associated with increased *pfmdr1* copy number was greater during the period 1996–2001 compared to thereafter for both artesunate (Pre: 2.1 *vs.* Post: 1.4 fold, *p* = 0.007) and mefloquine (Pre: 3.5 *vs.* Post: 2.6 fold; *p* = 0.076). In contrast there was no change in the risk of treatment failure associated with increased copy number over the study period.

## Discussion

The rapid development of mefloquine resistance in Tak province on the Thai-Myanmar border in the early 1990s led to the introduction of the three day combination of artesunate and mefloquine (MAS_3_) as first line treatment for uncomplicated *P. falciparum* malaria in 1994 in the camps for displaced persons. Despite the prevailing high levels of mefloquine resistance, cure rates increased to more than 90% and MAS_3_ had a spectacular impact on the incidence of *P. falciparum* malaria cases in the camps [19]. In vitro susceptibility of *P. falciparum* isolates to mefloquine improved significantly until 1999, attributed to the eradication of the more mefloquine resistant “strains” and the return of more mefloquine-sensitive “wild-type” parasites [23]. This was explained by the considerably reduced transmission advantage of mefloquine-resistant parasites when treated by MAS_3_, which are less fit than their mefloquine-sensitive counterparts [28], and consequent repopulation by “wild type” parasites from Myanmar (which there is little or no drug pressure) [26].

Over the subsequent 13 years of continuous MAS_3_ deployment the cure rates assessed at day-42 remain well above 90%. However despite this unprecedented level of sustained efficacy over the 13 year period and a significant decline in the prevalence of falciparum malaria on the Thai side of the border, there are some worrying trends. The in vitro susceptibility of *P. falciparum* isolates to artesunate has now returned to the levels observed in 1995, although the magnitude of this change is small (+/−4 nM) and within the accepted drug sensitivity levels. Mefloquine susceptibility has fluctuated widely, and its association with the *pfmdr1* copy number seems to have changed over the years. These changes in antimalarial activity in vitro have not been associated with a sharp reduction in cure rates, as happened when mefloquine was used as monotherapy [7], [19]. The changes in artesunate susceptibility could be related to the increases in *pfmdr1* copy number [25] although the patterns are dissimilar, and not well correlated with changes in mefloquine susceptibility. Over the last 4 years, there has been no further increase in the proportion of isolates with increased *pfmdr1* copy number, and yet there are indications that artemisinin efficacy has declined as evidenced by an increase in the parasite clearance time. The decrease in in vitro susceptibility to artesunate after 2002 could not be explained by mechanisms associated with mefloquine resistance (predominantly *pfmdr1* amplification). The correlation between artesunate and mefloquine IC_50_s evident before 2002 was no longer evident afterwards. This suggests the emergence of a new factor affecting artesunate susceptibility, which could include either novel mutations within the *pfmdr1* gene, increased gene expression or alternate genetic events.

The changes in the early in vivo parasitological responses to MAS_3_ are more concerning. Rapid parasite clearance is the pharmacodynamic hallmark of artemisinin and its derivatives. This is associated with reduced gametocyte carriage compared with other antimalarial drugs. There has been a small but significant slowing of parasite clearance, reflected by the increasing proportion of patients with parasitaemia persisting on the second and subsequent days following the start of treatment. Up until 2001 less than 7% of patients were parasitaemic on the second day of follow-up; however this figure has more than doubled in recent years. The delay in parasite clearance has been associated with a small increase in treatment failure rates, presumably reflecting a slight reduction in initial parasite reduction ratios, which results in a greater number of parasites for mefloquine to remove in the third and subsequent post treatment replication cycles [9]. Slowing of parasite clearance was associated with increased gametocyte carriage, and therefore presumably increased transmissibility of the more drug tolerant phenotype. This is an important public health point as the marked effect of artemisinin derivatives in reducing gametocyte carriage is an important contributor to their beneficial effects in malaria transmission control. Increased rates of gametocyte carriage were a harbinger of sulfadoxine-pyrimethamine resistance in Southern Africa, before any discernable effect on cure rates [29]. The dissociation between in vitro and in vivo observations suggests that the conventional in vitro approach to susceptibility testing may not be appropriate for evaluating artesunate tolerance. These are very early signs detectable only in this site, with the largest and most detailed longitudinal experience of antimalarial drug responses in the world. Several different factors affect cure rates. Young age of the patients, pure *P. falciparum* infections rather than infections mixed with *P. vivax* on admission, a parasitaemia over 40,000 parasites per µl on admission and site have been found to be independent risk factors for treatment failure following MAS_3_ [18]. However the temporal trends remain significant even after controlling for all these factors. The efficacy of the 3-day MAS regimen falls when the level of mefloquine resistance increases such that drug levels remaining after artesunate has been eliminated are not sufficient to eliminate completely the residual parasites. Mefloquine resistance arises readily as a result of gene amplification [25]. Drug pressure, and therefore the risk of selecting mefloquine resistant parasites results from increasing drug use, inadequate regimens, and from unprotected monotherapy [30]. Mefloquine has been used scantily within Myanmar. The 2-day regimen that was used by the Thai national programme was instituted in the early years of the combination therapy, based on non-PCR adjusted cure rates at 28 days of follow-up [31] and on the assumption that adherence to a two day regimen would be better than that with a three day regimen [3]. However with a two-day regimen, blood levels of artesunate cover one parasite asexual cycle only, so residual numbers of parasites left for the mefloquine component to remove are up to four orders of magnitude higher than with the three day regimen. This would be expected to increase the selection pressure for the emergence and spread of mefloquine resistant strains [32]. Drug pressure may have been further aggravated by the lower mefloquine dosage (15 mg/kg) used in monotherapy in 62 out of the 76 Thai provinces until recently [6]. However the inconsistent trend in the in vitro susceptibility assessments of mefloquine and the lack of any shortening in the interval to recrudescence argues against a major decline in mefloquine susceptibility.

Despite some worrying signs, which could only have been detected by these very large prospective studies, the 3-day mefloquine-artesunate combination is still more than 95% effective in Tak province, 13 years after its introduction in the camps for displaced persons. Considering that resistance to mefloquine monotherapy had reached very high levels before this combination was deployed, and that surrounding areas deployed different dose regimens, its longevity is remarkable. Our data suggest that changes have occurred in the prevalent *P. falciparum* population resulting in small but significant increases in parasite tolerance to the artesunate component of MAS_3_. These are considerably less in magnitude than those reported recently from Western Cambodia [33] but they may be the harbinger of much higher levels of artemisinin tolerance. Although *pfmdr1* amplification is a contributor to artemisinin susceptibility, the main cause of this reduction in artesunate susceptibility is not known. Of particular concern is the increased transmissibility of these tolerant parasites. The changes are small, but they justify a very close monitoring of the susceptibility of *P. falciparum* in this area, and in particular vigilance to detect early emergence of higher levels of artemisinin resistance. The development of resistance to artemisinin and its derivatives would be a global disaster for malaria control as current treatment regimen are dependant on this class of antimalarial drugs.
